# Looking under the veil: Challenges faced by people with disabilities in cross-border entrepreneurship

**DOI:** 10.4102/ajod.v9i0.645

**Published:** 2020-09-02

**Authors:** Keresencia Matsaure, Agness Chindimba, Felistas R. Zimano, Fayth Ruffin

**Affiliations:** 1 Mufakose Mhuriimwe High School, Harare, Zimbabwe; 2 Deaf Women Included & Centre for Special Needs Education, Great Zimbabwe University, Masvingo, Zimbabwe; 3 Department of Human Resources – PPA, Great Zimbabwe University, Masvingo, Zimbabwe; 4 School of Management, IT and Governance, University of KwaZulu Natal, Durban, South Africa

**Keywords:** PWDs, disability entrepreneurship, PWD’s empowerment, trade facilitation, cross-border trade

## Abstract

**Background:**

Cross-border entrepreneurship is one source of livelihood that is transforming people’s lives, especially those with limited resources and educational qualifications and those in need of supplementary earnings to complement meagre formal earnings. However, despite strides made to make this avenue worthwhile, this Zimbabwean study shows that hidden hindrances still persist from procedural and structural barriers from road entry point management systems. To people with disabilities (PWDs), the impact of these hidden barriers is severe to the extent of obstructing their optimum progression into cross-border entrepreneurship.

**Objectives:**

This article sought to interrogate some veiled challenges in border management systems affecting PWDs’ quest to venture into cross-border entrepreneurship. This angle has, to this end, been timidly addressed as most organisations and legislation have concentrated on making things work for the majority of the populace.

**Method:**

Qualitative phenomenological method in which researchers’ lived experiences, review of literature, ideas and opinions is complemented by secondary survey data from a road entry point management system study in the Zimbabwean setting.

**Results:**

Cross-border entrepreneurship has potential to transform people’s lives: 1) road and border management systems’ procedural and structural complications present hidden challenges impeding PWDs’ entry and optimum participation in cross border entrepreneurship, 2) people with disabilities are not automatically dependents; in fact, most have dependents looking up to the, 30 social construction of disability persists and must be curbed and 4) there is a need to institute a ‘stakeholders triad approach’.

**Conclusion:**

The existing road entry points’ management systems are not informed by considerations from PWDs, hence the existence of hidden challenges. Cross-border entrepreneurship can open significant livelihood avenues to PWDs. A stakeholders ‘triad-approach’, proposed herein, can solve some of the policy discrepancies as it recommends utilising inputs from PWDs, research and policy-makers.

## Introduction

Small to medium entrepreneurship (SME) is emerging as a viable source of livelihood and large-scale empowerment. In Zimbabwe, the springboard for most entrepreneurs at this level is in the cross-border trading (Muzvidziwa 1998; Zimano & Ruffin 2017). This brings to the fore the issue of movement of people and cargo across national frontiers and the associated requisites. It is an acknowledged and established fact that most small-scale cross-border trade is done by road, which in Zimbabwe is by and large to the adjoining countries: Zambia, Mozambique, South Africa and Botswana, whilst outside these adjoining territories, SMEs occasionally travel as far as Namibia, the Democratic Republic of Congo (DRC), Tanzania, Swaziland, Lesotho, Malawi and Kenya by road (Tawodzerwa & Chikanda 2016). In all these endeavours, traders encounter impediments associated with road entry point management systems (REPMS) as they cross borders. Impediments emanate, *inter alia*, from systems associated with entry points’ infrastructure (soft and hard) as well as procedural requirements. In addition, Muzvidziwa (1998) established an array of challenges that include high crime and theft of cash and goods. These challenges have been so persistent that they are slowly becoming inherent aspects of cross-border trade. This article submits that it is possible to eliminate these challenges for people with disabilities (PWDs) if policy-makers and implementers attend to some fundamental infrastructural and procedural facets of REPMS (Zimano 2017).

The approaches in addressing REPMS challenges adopted by countries seem to be ignorant of the impact of the existing REPMS on PWDs. Of late, much effort has been done revamping systems of their weaknesses culminating in adoption of ‘healthy’ infrastructure and procedures in most cases with the intention of improving passage for all. However, despite progress made towards empowerment, the world is leaving some groups behind (Hanass-Hancock et al. 2017). By so doing, various sections of the society remain excluded from mainstream activities. Whilst most developments in REPMS have eased movement of people from one country to another, there remains a lot to be done for PWDs.

To date, PWDs still face a myriad of prejudices in different commonplace experiences fuelling their exclusion in activities that are of economic value (Jaeger & Bowman 1974; Quarmby 2018. For example, Nuwagaba (2016) laments and exposes the inaccessibility of microfinance services by PWDs in Uganda because of policy and practice discrepancies. The failure to access microfinance services may perpetuate exclusion of PWDs from main economic activities. This is one case amongst several others that will be presented in forthcoming sections that justify the need for revamped multidisciplinary and stakeholder approaches to solving this and other problems faced by PWDs in this sector.

By highlighting the importance and extent of cross-border entrepreneurship in modern day life, this article situates the importance of REPMS into this debate. It brings out issues of hidden disablers as core elements of discussion showing that some hindrances that PWDs are facing are not naturally occurring because of their physical disabilities but they are a result of omissions or commissions in design and implementation of REPMS infrastructure and procedures. A study on various Zimbabwean REPMS brings secondary insights that are used herein to expose those not so obvious border-systems-related challenges affecting the progression of PWDs into cross-border entrepreneurship. Most PWDs are found in developing countries (Quarmby 2018; Zimano & Ruffin 2018). As such, using the Zimbabwean case can give significant insights into this phenomenon. With the understanding of the social construction theory, this article exposes ways in which the society is reinforcing some hidden challenges at border points and eventually perpetuating the exclusion of PWDs from participating in cross-border entrepreneurship. The social construction theory brings out ideas that challenges society to stop automatically viewing PWDs as ‘dependents’ as this takes away their independence militating against their empowerment. Some societies consider disability as a curse or punishment from God or ancestors (Ngubane-Mokiwa 2018). Such views reduce effectiveness of initiatives whilst marginalising PWDs (Cleaver et al. 2018). Besides, most PWDs have social responsibilities and desires to shoulder that there is a need to facilitate their optimum inclusion in all facets of life. By viewing them as automatic ‘dependents’, the society negates its duty of empowering PWDs to be independent in spite of impairments. Accordingly, this article brings together issues in REPMS that manifest as hindrances to PWDs entrepreneurship to expose how the society has created the barriers by failing to recognise the need of all the various stakeholders interacting with REPMS in their economic activities.

## Significance of the study

The study is of importance in aiding the empowerment agenda to enable optimum participation of PWDs in cross-border entrepreneurship. The study brings out findings on existing REPMS to expose the hidden challenges affecting PWD. This study also adds to the body of knowledge of the general structural and procedural hindrances in movement of people across borders and possible ways to alleviate them.

## Methodology

The design used herein combined review of literature and qualitative phenomenological approach in the Zimbabwean REPMS setting. Crucial to this research is the researchers’ lived experiences in which one of the researchers is a person with disability and PWDs empowerment activist. This brought in the aspect of qualitative phenomenology to this study. Phenomenological study is one that capitalises on lived experiences (Hosken 2018). The study also utilised secondary surveys and observations findings from an earlier study on four border points on Zimbabwe’s territorial borders (Plumtree, Chirundu, Beitbridge and Nyamapanda).

These borders were picked at random after first stratifying the 16 border points on Zimbabwe’s territorial lines by their geographical location. Each of the four locations thus contributed one border to the study.

### Ethical consideration

Ethical approval to conduct the study was obtained from the Research Ethics Committee of the University of KwaZulu-Natal (ethical clearance number: HSS/1165/015D).

## Theory and literature findings

Although the main thrust of this article revolves around REPMS and entrepreneurship, this part tackles the concept of disability. This lays the foundation into rationalising this whole article by attempting to answer a number of sub-questions, such as:

- What informs policy makers to make some decisions without considering PWDs’ plight?
- What causes society to offer mere sympathy rather than empowerment to PWDs?
- Are all PWDs dependents?
- How do social labels impede empowerment of PWDs?

There is popular perception that PWDs are dependents (Barnes 2000; Oliver 1989). This is the first issue that must be addressed in PWDs’ empowerment. Dependency implies that one is not able to assist own self in some or all everyday tasks leading to reliance on others (Oliver 1989). In as much as PWDs have physical challenges impeding their optimum functioning, they have desires whilst most also have responsibilities. The adult with a family, for example, has to carry out all duties and responsibilities expected of a parent to their children. Thus, PWDs equally want to get for themselves and be able to provide for their children all the basic needs as identified by Abraham Maslow’s hierarchy of needs theory. These needs are, in ascending order, physiological, safety, love/belonging, esteem, self-actualisation and self-transcendence (Tay & Diener 2011). All these come with associated expenses. As such, empowerment must give one the capacity and ability to meet daily life expenses. Examples of basic daily expenses are listed in Table 1.

**TABLE 1:** Day to day expenses associated with human needs.

Human needs	Functions related to the need	Associated basic activities and expenses
Physiological needs	Air, water, food, clothing and shelter	Utility bills, food and clothing, rentals and accommodation
Safety needs	Personal security, financial security, health and well-being security, safety security, etc.	Home security systems, medical insurance payments, financial investments, etc.
Social belonging	Friendship, intimacy, family, etc.	Social club fees, etc.
Esteem	Those that eliminate inferiority by giving the sense of contribution	
Self-actualisation	Getting to being the best of one’s potential	Children’s school and other fees *inter alia*
Self-transcendence	Altruism and spirituality	Tithes and other offerings as stipulated in various religions

To be able to meet these daily financial demands and more, PWDs must be empowered to fend for themselves in their conditions. This means alleviating the barriers to their participation in available opportunities. United Nations (2017a:1) in the *Convention of the Rights of Persons with Disabilities and Optional Protocol’s preamble* item (e) recognises disability as:

An evolving concept and that disability results from the interaction between persons with impairments and attitudinal and environmental barriers that hinders their full and effective participation in society of equal basis with others. (p. 1)

This means one’s abilities are reinforced or limited by the attitudes that a community holds and the conditions that the environment offers. A community with retrogressive attitudes can push someone’s abilities down whilst a progressive community can avert the loss of abilities. The same applies to the environment – the availability of an enabling environment can go a long way in curtailing what one can and cannot do. The issue of the ‘dependents’ tag is also linked to these attitudinal barriers. The ‘dependents’ tag, indicated above, comes with several consequences. It affects PWDs’ psychological disposition as biological factors and social factors interact in creation of a disability (Wendel 1996). This is a situation whereby one’s mind is skewed into believing they cannot fend for themselves leading to a dependency syndrome. Both PWDs and their families can hold expectations to charity which reinforce their exclusion (Nuwagaba & Rule 2016). The ‘dependents’ tag also culminates in social construction of disability. The social arrangements and beliefs can make a biological condition more or less relevant to almost any situation (Andrews 2012; Wendel, 1996). This means the extent of a biological condition depends on the beliefs that a society holds. Therefore, the dependency tag given to PWDs is purely a creation of modern industrial societies’ policies (Oliver 1989).

Finally, the ‘dependents’ tag leads to the creation of several hidden hindrances as policy makers fail to prioritise the plight of PWDs in their planning because once PWDs are classified as having little value then very little will be done to provide them equal access (Jaeger & Bowman 1974). In this way, the context will be set for the creation of disability as the interaction between societal attitudes and available infrastructure shape disability (Swartz & Schneider 2006). This occurs because PWDs will be falling outside the category of key consumers of facilities or policy provisions. Most developing countries, for example, use charity or medical models of disability – models which view PWDs as sick or childlike people who have to be taken care of (Rugoho & Chindimba 2018). Policy-makers concentrate on empowering those without disabilities with the perception that these in turn shoulder the responsibility of looking after PWDs – ‘dependents’. As such many policies targeting PWDs are rooted on the assumption that they will get personal assistance from family members, children or spouses (Barnes 2000). This oversight is evident in Zindiye, Chiliya and Masocha’s (2012) analysis of ‘targeted support’ in which they exposed extensive government support towards ‘cluster-based development’, ‘gender dimension in development’, ‘youth development’ and ‘rural focus’.

Although PWDs fall into all those groups, they should have been afforded their own category as initiatives in the said groups will not necessarily address their plight. Such prejudices reduce their levels of access in society (Jaeger & Bowman 1974). This shows how, by itself, the ‘dependents’ tag blindfold those PWDs and the society from reality. There is a need to work towards eliminating that. Once PWDs get classified as people of value in society, then issues of equal access become social concerns (Jaeger & Bowman 1974).

However, the removal of the ‘dependents’ tag does not come through rhetoric. The Convention on the Rights of Persons with Disabilities and Optional Protocol covers everything from their rights through to empowerment issues. For them to be able to fully enjoy their rights, there is a need for awareness and provisions of empowering strategies. Empowerment comes through provision of enablers to ensure the independence of PWDs. One such, as listed in the convention is entrepreneurship. The world over, entrepreneurship has emerged as a viable source of livelihood (Tawodzerwa & Chikanda 2016; Zimano & Ruffin 2018). In Zimbabwe, people engage in several entrepreneurship ventures but cross-border entrepreneurship seems to be top amongst the most popular (Muzvidziwa 1998; Tawodzerwa & Chikanda 2016; Zimano 2017). In order to ensure that PWDs also actively venture into this source of livelihood, there is a need to remove the disablers in the cross-border entrepreneurship environment.

### Cross border entrepreneurship as empowerment

Cross border trading is changing people’s lives for the better (ESCAP 2014; Muzvidziwa 1998). The practices of cross-border movements in general and cross-border trading in particular are not a new phenomenon. In Africa, this practice dates back to times before the arrival of the colonisers as cross-border movements can be traced back to the *mfecane* period in places like South Africa and Zimbabwe (Hungwe 2012). Most cross-border entrepreneurs start plying the trade informally with recruitment happening in friendship networks. Research has shown that 85% of people in cross-border entrepreneurship were initiated by friends whilst the remaining 15% is by kin (Muzvidziwa 1998). There is evidence showing that a lot of people who venture into this type of livelihood eventually take it as their lifelong source of livelihood. Some respondents to a survey on cross-border trade complementing this study indicated that they had been in the trade for more than three decades (Zimano 2017). The high numbers of people taking up this source of livelihood testify to its viability both to the country and at household level (Titeca & Kimanuca 2012). In order to appreciate what those failing to venture into this trade are losing out, there is a need to unpack some of the benefits accruing to those in the trade.

Cross-border entrepreneurship and any other informal entrepreneurship in general, is less capital intensive and normally start informally (Marunda & Marunda 2014; Zindiye et al. 2012). This can be a starting point as majority of PWDs live in conditions of poverty (United Nations 2017a). Without significant capital, one can start by buying and selling very few items. One can also access viability of desired venture before committing too many resources. One can also choose to venture into entrepreneurship at individual level (Marunda & Marunda 2014). That is why a lot of low income earners have found it viable (Muzvidziwa 1998; Titeca & Kimanuka 2012). It can also be done through self-financing. Self-financing has several advantages anchored by the independency entrepreneurs get. It eliminates complex partnerships, allows one to venture into aspirations of their desire whilst proceeds go directly for personal use and quitting can be done without strenuous procedures (Cornwall, Vang & Hartman 2009).

There are no deterrent educational qualifications requirements for entry. Some survey respondents indicated having only elementary education (Zimano 2017). This means those people with lower educational qualifications can utilise this to their advantage. The informal sector significantly contributes towards poverty alleviation and employment creation because of its ability to absorb unskilled and semi-skilled workforce who would ordinarily be left out of formal employment (Chingwenya & Mudzengerere 2013). A lot of PWDs in developing countries fail to secure employment because they do not have sound educational qualifications (Naami 2015). In such instances, venturing into entrepreneurship allows one to utilise skills and knowledge that falls outside basic educational qualifications. A lot of people are making a living travelling to other countries to engage in activities like hair plaiting, laundry services, menial jobs and seasonal farm working.

There are public and private bodies assisting those venturing in cross-border entrepreneurship. In Zimbabwe, several opportunities are availed through the Ministry of Small to Medium Enterprises and Cooperatives Development. This was established in 2002, as the then Ministry of Small to Medium Enterprises showed the government’s realisation of the sector’s growing importance (Chivasa 2014). This ministry promotes and coordinates financing schemes for SMEs; it also facilitates linkages, and provides skills and management training support. These efforts are complemented by several private players through loan facilities, educational programmes and social support. Examples include social clubs, church organisations, well-wishers and community out grower schemes amongst others. In Zimbabwe, the government deliberately endeavours to motivate the growth of SMEs through tax relief as they are not subjected to full rates of tax whilst tax rebates and discounts are extended to most of their acquisitions (Zindiye et al. 2012). Clearly, venturing into self-employment and entrepreneurship is being made relatively easy.

Although self-employment is categorised as vulnerable employment by the United Nations (2017b), it is better than staying without any productive economic engagement. Employment is characterised as being vulnerable if it falls into low income bracket, does not give one job security and also lacks job-related benefits (Naami 2015). Nevertheless, self-employment gives satisfaction and happiness to PWDs as compared to their counterparts in formal employment as they get to make their own decisions (Marunda & Marunda 2014; Naami 2015). This is consistent with the human needs discussed in earlier in this article. This satisfaction is required for the psychological stability. Self-employment also comes in handy by providing flexible working hours for PWDs (Naami 2015). They can properly plan their work taking into consideration their executing capacity. In the context of cross-border entrepreneurship, self-employed PWDs can schedule their travel taking into consideration weather patterns and other factors such that they travel when favourable to them.

Cross-border entrepreneurship can be one’s form of employment from early adulthood years up until later years of life. Survey respondents median age was 31 years (inter quartile range was 19 to 60 years) (Zimano 2017). This is because there is readily available mentorship from established entrepreneurs. Even in the absence of basic mentorship, one can learn the basics of the trade through observing and imitating (Muzvidziwa 1998). The various items that people trade in allow people to adjust and remain in the trade until old age. The young and ambitious ones can venture into fast selling goods and travel far and wide in the region. Those getting older can adjust their trade to suit their capabilities and limit travelling.

There is a ready market for cross-border entrepreneurs’ goods and skills (Titeca & Kumanuka 2012). This is because entrepreneurs cover the gap between the consumers and the industries. They help by breaking bulk by delivering just the right quantities to meet the consumer’s needs. Cross-border entrepreneurs take wares to other countries for sale whilst bringing in goods from other countries into the local market (Tawodzerwa & Chikanda 2016; Titeca & Kimanuka 2012). In so doing, they bridge the gap in the distance for those who are formally employed and who might not have time to travel. The informal cross-border courier business, *malaitsha or magumhagumha*,^1^ is also another thriving source of income for enterprising people.

The cross-border entrepreneurship provides supplementary income for enterprising formally employed individuals. This is because of the flexibility it offers in terms of working hours. Some people in formal employment travel to neighbouring countries over the weekend to get stock. They then go by ‘handbag’ retailing in which they move around with small wares for sell at their workplace, in their neighbourhoods and places of worship. As such, even PWDs need to go an extra mile even in cases where they get social security grants from governments. These grants are usually insufficient to meet all their basic needs (Ned & Lorenzo 2016).

To this end, it is clear that cross-border entrepreneurship is less capital intensive, open even to people with lower qualifications, provides supplementary income for families and exposes players to new markets and knowledge amongst several other advantages. These advantages are most likely the rationale behind calls to help PWDs to venture into entrepreneurship enshrined in Article 27(f) of the Convention on the Rights of Persons with Disabilities and Optional Protocol as it talks about the promotion of self-employment opportunities, entrepreneurship, venturing into businesses and cooperatives development (United Nations 2017a). However, these benefits do not come on a silver platter. There are hindrances that have militated against its growth and sustainability. Several cross-border SMEs indicated their desire to formalise their trade but cited procedural red tapes as key hindrances (Zimano 2017). This has seen most opting for informal routes that are risky – some end up losing their goods, health and even life in the process (Titeca & Kimanuka 2012). Pertinent to the challenges cited were the problems associated with clearing goods on countries’ borders. This means there is a need to give undivided attention to the place of REPMS in the empowerment debate in order to get an understanding of how they have led to the creation of obstructions for PWDs. This is because outside the social security grants they sometimes receive from government, there is still limited economic empowerment for PWDs (Ned & Lorenzo 2016).

### The place of road entry point management systems

The challenges faced by people in cross-border movement of goods and trade are either tariff barriers (TBs) or non-tariff barriers (NTBs). Tariff barriers are wide and varied taxes imposed on imports in order to protect local industries by making imports more expensive than domestic products (Farlex Financial Dictionary 2012). They include *ad valorem* (tax assessed on merchandise), duties (charged by weight, volume, length or any other unit), compound interests, alternative duties, value added tax amongst other things (Manzella 2001). On the other hand, NTBs encompasses restrictions emanating from prohibitions, conditions or market specifications that complicate the importing or exporting of products (COMESA-EAC-SADC n.d.).

However, even though TBs create hindrances for cross-border SMEs because of their feeble financial muscle and meagre technical know-how, this article is not to prioritise TBs issues. This is because TBs do not selectively affect people because of their physical abilities. The thinking is that anyone with the financial strength (through self-financing or loans) and technical knowhow (personal or through consultations) can properly register a company and competitively operate above board regardless of being with or without disabilities. As such, the focus from now will be on NTBs. These have a selective impact on people depending on one’s physical abilities. Non-tariff barriers, unlike TBs, are usually difficult to quantify or measure and are often hidden (Manzella 2001).

Non-tariff barriers are factors, besides taxes, that impede the flow of trade (Xiong 2012:13). They are those things that are not pronounced in monetary terms. They revolve, mostly, around procedural and infrastructural issues that disturb the smooth cross-border movements of goods and traffic. For itself, there is a need to understand the REPMS in use before one can properly appreciate the prevalence of NTBs and subsequent hindrances affecting PWDs. In this article, the focus is limited to procedural and infrastructural NTBs occurring in the Southern Africa Development Community (SADC) entry points with an impact on PWDs as evidenced in the case of Zimbabwe REPMS. It is also needed to appreciate that disabilities occur because of a wide and varied reasons. ‘Disability can manifest as a physical or cognitive issue coming from a range of factors – genetics, accident, external circumstances or advancing age’ (Jaeger & Bowman 1974:6). This should open readers’ minds to understand the far-reaching impact of prejudices affecting PWDs.

There are two types of REPMS in SADC: the one-stop-border-post (OSBP) and the two-stop-border-post (TSBP). The OSBP system is in use at Chirundu – the border between Zambia and Zimbabwe (Kassee 2014:105). Under this system, vehicles and travellers crossing borders go through entry and exit formalities in one facility eliminating double stoppage and duplication of procedures (WTO 2011). Before the OSBP was introduced at Chirundu, trucks took two to three days to be cleared. This was reduced to 2 h by OSBP system (Zimano 2017). The main thrust for such an initiative is trade facilitation through reducing time at the border and cross-border transactions. Trade facilitation refers to the capacity for goods to be moved across national borders (Hewitt & Gillson 2003). The rest of the entry points on Zimbabwe’s territorial borders use the TSBP in which transporters and travellers stop and get exit clearance as they emigrate before making another stop for immigration formalities into the neighbouring country. Whether OSBP or TSBP, one goes through procedural issues and also interact with the facility’s infrastructure. This is where, because of omission or commission as mentioned earlier, the procedural and infrastructural NTBs emanate form.

Procedural NTBs relate to the way things are done. On the other hand, infrastructural NTBs pertain to the hindrances from interaction with the hard and soft components of the entry point. Infrastructure in place contributes to the experience of living with disability (Swartz & Scheider 2006). Zimano’s (2017) study established several such NTBs occurring on Zimbabwe’s entry points as shown in Table 2.

**TABLE 2:** Procedural and infrastructural non-tariff barriers on entry points.

OSBP	TSBP
**Procedural NTBs** Law manpower Absence of sound legal framework Absence of strong commitment to ethical practices and insincerity towards harmonisation Inadequate skilled manpower with border management skills	Less interaction amongst agencies Border efficiency management skills not implemented Procedures not streamlined Absence of clear objectives on ICTs usage to ensure optimum use Entry points working less than 24 h a day Limited use of sound support structures like the cargo pre-clearance systems Lack of enough border management skills
**Infrastructural NTBs** Incompatibility of systems and absence of systems interface Hard infrastructure not informed by soft infrastructure OSBP not implemented at most border posts Network system not allowing interconnectivity and systems lacking interface OSBP not existing in a series along the whole corridor	Absence of single window system Poor road networks around the border areas Absence of physical structure to separate outward bound from inward bound traffic to avoid mixing Systems not fully automated

*Source*: Zimano, F.R., 2017, ‘Road entry point management systems and regional integration: The case of Zimbabwe’, Unpublished Doctoral thesis, viewed 01 June 2018, from http://researchspace.ukzn.ac.za/handle/10413/15338.

ICT, information and communication technology; NTBs, non-tariff barriers; OSBP, one-stop-border-post; TSBP, two-stop-border-post.

Non-tariff barriers listed above affect all entry point users; living with disability or not. Some of the NTBs result in unprecedented clearance delays. This comes with its associated vices like loss of goods, physical body strain leading to fatigue and corruption as people try to use unorthodox means to hasten their clearance amongst several other challenges. However, over and above this, by their negative impact, the NTBs create hidden disablers for PWDs. Most of these issues are consistent with those highlighted under ‘accessibility’ issues; Article 9, in the *Convention on the Rights of Persons with Disabilities and Optional Protocol* (United Nations 2017a).

Accessibility issues entail the rights to equal participation in ways free of mental or physical constraints (Jaeger & Bowman 1974). ‘Access can be viewed in terms of physical access (e.g. to objects) and intellectual access (e.g. to ideas and information)’ (Jaeger & Bowman 1974:20).

The procedural NTBs listed above culminate in delays. Firstly, there is the issue of multiplicity of players and duplication at the entry points. According to Widdowson and Holloway (2011):

Contemporary border management reflects a complex interplay between a variety of actors in international trade, both across government through its public sector agencies and between government and the private sector. (p. 95)

Various ministries are involved in REPMS operations, including those involved in revenue collection, animal and plant quarantine, transportation and vehicle inspections, immigration and security (Zimano 2017). In this case, the procedural NTBs are reinforced by infrastructural NTBs. The absence of a single-window clearance system means players operate from different clearing points. Players usually lack interface such that one moves to and fro several counters before getting cleared. When clearing at Beitbridge one has to pass through the police department which is situated in its area before reporting to customs (Munyanyi 2015). The absence of single-window clearance systems with sound interface presents challenges to PWDs, especially those with mobility limitations. These often use wheelchairs or clutches (Visagine et al. 2016). Offices that require people to move from one office to another complicate everyone’s abilities but worsen the plight of PWDs.

There is evidence that the employees manning borders in different departments lack or choose not to implement contemporary border efficiency management skills (Zimano 2017). Even though significant investments by governments and the development community have been made into border management reform and modernisation, there will be no changes to performance unless the changes in infrastructure are accompanied by the adoption of modern ways of managing the borders (Zarnowiecki 2011). There is generally poor organisational culture culminating in low morale leading to a lack of urgency in the way clearance is done (Zimano 2017). This often results in clearance delays resulting in holding bays overcrowding. This is why most borders are marred with crowd related problems in which law enforcement agencies have sometimes resorted to using force. This is not favourable to PWDS who might not be able to withstand the chaotic environment without getting injured and losing their goods in the process.

Another procedural NTB listed is that all but one, Beitbridge, entry points do not operate 24 h. The relatively busy entry points like Plumtree and Chirundu only get seasonal waivers to operate 24 h. With the absence of proper holding areas, such operational hours present challenges to PWDS especially those with the albinism condition. Albinism is an inherited condition in which ones’ system does not produce melanin thus becoming prone to sunburns and subsequently skin cancers (eds. Parker & Parker 2003). Almost all the Zimbabwean borders, save for Forbes and Espungabera Manicaland, are in climatic regions 4 and 5.

Zimbabwe’s climatic regions 4 and 5 receive annual rainfall below 600 mm and are characterised with severe dry spells (USDA 2017). To make matters worse, the most relevant entry points for cross-border entrepreneurship: Plumtree, Beitbridge and Chirundu are in climatic region 5 which receives the most extreme hot temperatures than the rest of the country. The initiative to have the Beitbridge entry point operating 24 h is good because people with skin pigmentation disabilities can plan their journeys and capitalise on evening hours when temperatures will be a bit favourable to them. However, this will only work to their advantage if there are no delays in clearance at the borders. With the rest of the entry points that do not operate 24 h, travellers have to brave the daytime extremely high temperatures. This, coupled with the absence of properly air temperature conditioned holding halls, deter the participation of people with albinism in cross-border entrepreneurship as they cannot risk exposing their skin to such conditions.

The low performance and utilisation of information and communication technology (ICTs) also present infrastructural NTBs on entry points. Embracing the e-business models, as well as putting measures in place to ensure that the ports operate effectively can lead to effective trade performance in the SADC region (Makochekanwa 2013). Information and communication technology encompasses both physical and intellectual access (Jaeger & Bowman 1974).

However, there is evidence that systems lack compatibility, network systems do not allow total interconnectivity, the systems are not fully automated whilst systems also lack interface (Zimano 2017). All these challenges erode the benefits of ICTs that should be alleviating most of the woes at entry points. A good number of PWDs can operate and own wireless devices. These gadgets offer substantial cross cutting opportunities from independence, social participation and education right up to basic security (Bornman et al. 2016). Such ‘technological advances can even eliminate a disability’ (Jaeger & Bowman 1974:6). By utilising mobile money banking systems available on most mobile service providers platforms, cross-border entrepreneurs reduce the risk of moving around with large amounts of hard cash. The poor interconnectivity wears down such benefits of paperless transactions. Paperless trading is also an effective way of reducing trade costs (ESCAP 2014). In cases of communication breakdown with the officials manning the borders from various departments, PWDs can contact their next of kin back home and get assistance over their mobile phones and various computer-based communication platforms. Computer technology has recognised potential to enhance PWDs’ ability to participate alongside wireless technology advances (Bornman et al. 2016; Mosito, Warnick & Esambe 2017). However, the absence of sound interconnectivity eliminates all this associated security and convenience reflexively creating hidden disablers for PWDs.

The proper use of signs, symbols and verbal cues at entry points is very limited. These researchers made a random check of the signage at entry points to great disappointment and confirmed Munyanyi’s (2015) observation that information centres are either rundown or unmanned. The most visible signs are only those for ablution facilities and prohibition signs. Most clearance halls nowadays utilise the overhead voice amplifier systems to issue supplementary regulatory messages without supplementary sign translations for people with hearing impairments. This shows an oversight as the deaf will not be able to get such messages. This shows a downside in the use of mechanised tools without extensive considerations resulting in deprivation for PWDs (Barnes 2000). In the absence of televised screens translating overhead voice notices, this group of people will remain in the dark. This is coupled by the fact that, besides the negative stigma already associated with hearing loss, people living with this type of disability inherently do not like to admit their having the condition and in most cases do not want to ask (Green, Maphosho & Khoza-Shangase 2015). This leads to communication breakdown. Once there is communication breakdown, there are chances that someone with hearing disabilities will act in a manner inconsistent with the procedures announced orally creating an environment for unhealthy confrontations and contradictions. In the case of those with visual impairments, the signs do not always come with supplementary voice such that they have to rely on asking other people.

Considering that an entry point is a meeting point for several people using several vernacular languages, the visually impaired person will have to go an extra mile to find those who speak their local language. Given the ever increasing undisputed importance of information, there is a need to ensure access for PWDs (Jaeger & Bowman 1974).

Another infrastructural challenge is that there is only one entry point under the OSBP system. The existence of only one OSBP in the SADC region leads to discordances in the flow of people’s movement. The OSBP system at Chirundu ensures fast clearance for travellers (WTO 2011). However, if these travellers and traffic are in transit, the fast clearance can culminate in problems at their next stop if the borders do not have the same clearing capacity. What it means is traffic will stay less time at Chirundu only to accumulate and stay longer at, for instance, Beitbridge. This is a challenge that has brought calls for the development of entry points in a given corridor to adopt similar and complementary REPMS (Zimano & Ruffin 2018). Once traffic gets piled at one entry point, the operating halls become crowded. This is worsened by weak queue management at entry points like Beitbridge (Munyanyi 2015). This results in noisy environments unfriendly to people living with some disabilities. Research has it that those individuals with hearing disabilities have difficulties communicating against noisy backgrounds (Brennon & Bally 2007). The prolonged stay waiting for clearance also leads to fatigue. This affects all road users but is worse for PWDs as they will not be able to withstand prolonged strain on their bodies.

Directly related to the efficiency of the operations at the entry points is the state of the road networks leading to the border points. Zimbabwe has a poor road network riddled by potholes and narrow sections. This makes travelling by road very uncomfortable for all and sundry. According to Watermeyer (2006), most PWDs fail to reach opportunities because of the absence of safe transport systems. The roads cause a lot of back pain to all travellers that anyone can imagine the severity to PWDs.

#### Envisioned interventions for the removal of the hidden hindrances

### The stakeholders’ triad approach

The challenges faced by PWDs because of REPMS presented in the theory and literature findings section are merely an eye opener.

**FIGURE 1:**
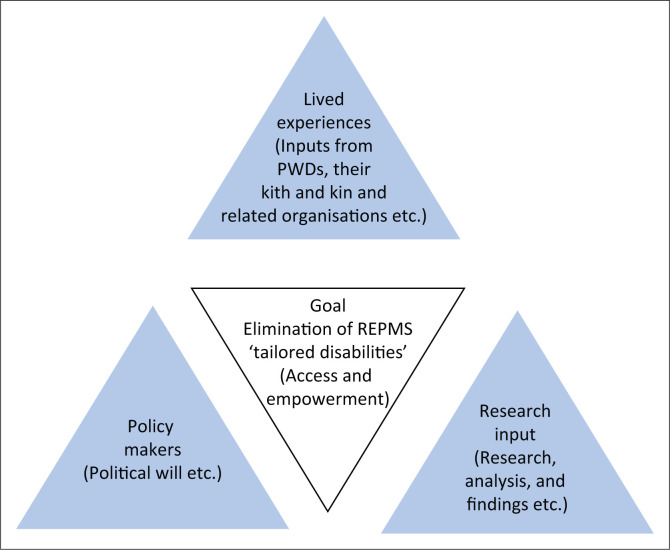
Stakeholders’ triad in the removal of road entry point management systems hidden disablers.

The centrality of the problem is that it is ‘very hard to understand disability if one has neither experienced a disability nor been close to someone else who has a disability’ (Jaeger & Bowman 1974:12). The best method, envisioned herein, is to engage PWDs, their kith and kin, and organisations working with them. There is a need to establish networking platforms and sector-based collaborations to ensure successful disability awareness and inclusion (Ned & Lorenzo 2016). These will take policy makers and researchers through their lived experiences giving a more comprehensive understanding of the problem beyond what this article can achieve. Once that happens there is need for a strong political will. A lack of political cohesion is a key hindrance in that it is the political will that informs the country’s actions (Peteris 2013). As such, the recommendations listed below will only materialise into tangible benefits if the missing voice of PWDs is brought in together with the political will in a triangulated approach. The triangle, shown in Figure 1, brings the work from academics, the voices of PWDs and the policy makers together for the greater good of the whole society as hidden factors hindering the empowerment of PWDs through cross-border entrepreneurship will be alleviated.

### Interventions for hidden disablers’ removal

Having brought out all the stakeholders’ place, a lot of initiatives will be proposed. In line with the barriers discussed in this article, the following interventions, summarised in Table 3, can be adopted as a starting point.

**TABLE 3:** Interventions to alleviate hidden disablers.

REPMS Identified challenges	Intervention	Impact to PWDs
1. Systems not fully automated 2. Network system not allowing interconnectivity and systems lacking interface	Fully embrace ICT to enhance smooth flow of information in various media forms	Remove communication breakdown challenges Reduce incidences of moving around with hard cash through the use of plastic and mobile money platforms
3. Absence of single window system 4. Limited use of sound support structures like the cargo pre-clearance systems 5. Procedures not streamlined	Harmonise systems and streamline procedures	Reduced mobility challenges as players will be housed under one roof Streamlined procedures lessen points of clearance reducing mobility challenges
6. Entry points working less than 24 h a day	Introduce longer opening hours preferably 24 h Introduce proper holding halls with air temperature conditioners	Reduced exposure to harsh weather conditions Reduce queues eliminating crowd related challenges
7. Poor road networks	Road rehabilitation	Reduced back pain and strenuous travelling experiences
8. Lack of enough border management skills 9. Absence of strong commitment to ethical practices and insincerity towards harmonisation 10. Inadequate skilled manpower with border management skills	Enforce the training and implementation of BEMS and increase manpower	Improved border-environment culture allows all to access services effectively

BEMS, border efficiency management skills; ICT, information and communication technology; PWDs, people with disabilities; REPMS, road entry point management systems.

As presented in Table 3, there are various methods that can be used to rectify the challenges PWDs facing raised in earlier sections. There is a need to upgrade and avail ICT systems at all border points. This will bring in the gains from ICT currently eroded by the absence of full automation. Automated systems will go a long way in enabling PWDs to use several online platforms to bridge any gaps in their interaction with various people at border points. The benefits that come with different weather conditions and times of the day to PWDs can be resolved by having border points operating 24 h. This means those people who struggle with hot or cold weather conditions can capitalise on the times they see best fit their conditions rather than being restricted to travelling during the day. The need to continuously empower the people manning borders to adapt to emerging challenges cannot be overemphasised as more contemporary border efficiency management skills (BEMS) have a potential of alleviating most of the challenges raised.

## Study limitations

This study is confined to experiences at Zimbabwean entry points. As such, experiences reported herein may be more pronounced or reduced because of other factors such as cultural and economic factors that are purely Zimbabwean. For future researchers, the use of entry points from other countries to bring comparative experiences can be useful to bring more insights on how other countries’ systems are enabling or disabling PWDs’ optimum participation in cross border entrepreneurship.

## Conclusion

This article has discussed hidden factors in border management systems affecting PWDs in their quest to venture into cross-border entrepreneurship. This angle has, to this end, been timidly addressed as most organisations and legislation have concentrated on making things work for the majority of the populace. People with disabilities have been, to date, widely viewed as dependents. The assumption being that empowering the able-bodied inadvertently caters for PWDs as the able would take care of PWDs. However, evidence has shown that this has culminated in the social construction of exclusion that disempowers PWDs. In this article, it has been argued that most PWDs are not dependents; in fact, most have dependents under their care. It has also been shown that cross-border entrepreneurship by road is a source of livelihood in Zimbabwe. This situated REPMS infrastructure and procedures in the debate showing how they can be a source of empowerment if properly constructed and implemented. On the other hand, this article has shown how the REPMS can result in some hidden hindrances impeding the active and productive participation of PWDs in the field of cross-border entrepreneurship if improperly constructed, maintained and managed.

What is required is to draw policy makers’ and PWDs’ attention to these pertinent issues in REPMS. It is the duty of all to see to it that PWDs get out of the ‘dependents’ brackets and go out to see how they can fit into the emerging empowerment frameworks. In so doing, they will add their voices based on practical experiences to issues raised in this article on the best way the existing REPMS can be dealt with to eliminate the hidden challenges. The policy makers must embrace these and more ideas to ensure that the total empowerment of PWDs and their participation in society becomes a reality. The creation of a working triad for key stakeholders will help bring together the lived experiences, the research findings and analysis, and the political will necessary for the comprehensive addressing of challenges in REPMS. Governments must take it upon themselves to deliberately speed up initiatives such as harmonisation, streamlining of procedures, implementation of BEMS, provision of friendly holding halls at entry points, utilisation of ICT products and rehabilitation of road networks amongst other things for the good of all and the direct empowerment of PWDs. These initiatives directly remove challenges linked to communication, mobility, strenuous travelling conditions and extreme weather conditions’ impact on skin, and travellers’ security amongst other things which emerge as imperceptible obstructions because of procedural and infrastructural commissions or omissions. Above all, the earlier all the stakeholders take these issues seriously, the earlier the arguments presented herein will make sense to all and the earlier their issues will be prioritised in mainstream policy considerations.

## Footnotes

1 Malaitsha/Magumhagumha are vernacular words used loosely to refer to people who do the informal facilitating and courier in cross border trade.
